# Insights into Phylogeny, Taxonomy, Origins and Evolution of *Crataegus* and *Mespilus*, Based on Comparative Chloroplast Genome Analysis

**DOI:** 10.3390/genes16020204

**Published:** 2025-02-07

**Authors:** Jiaxin Meng, Yan Wang, Han Song, Wenxuan Dong, Ningguang Dong

**Affiliations:** 1 Institute of Forestry and Pomology, Beijing Academy of Agriculture and Forestry Sciences, Beijing 100093, China; wangyannifp@baafs.net.cn; 2 College of Biological Sciences and Biotechnology, Beijing Forestry University, Beijing 100083, China; songhan@bjfu.edu.cn; 3 College of Horticulture, Shenyang Agricultural University, Shenyang 110866, China; wxdong63@126.com

**Keywords:** *Crataegus*, chloroplast genome, phylogeny, molecular dating, biogeography inference

## Abstract

Hawthorns (*Crataegus* L.) are widely distributed and well known for their medicinal properties and health benefits. Nevertheless, the phylogenetic relationships among *Crataegus* native to China remain unclear. Additionally, no consensus exists on the origin and evolution of *Crataegus*, and the relationship between *Crataegus* and *Mespilus* is is unclear. Here, we sequenced 20 chloroplast (cp) genomes (19 from *Crataegus* and 1 from *Mespilus*) and combined them with 2 existing cp genomes to investigate the phylogenetic relationships, divergence times and biogeographic history of *Crataegus*. Four hypervariable loci emerged from the newly sequenced genomes. The phylogenetic results indicated that the 14 Chinese *Crataegus* species analyzed clustered into two clades. One clade and the North American *Crataegus* species grouped together, while the other clade grouped with the European *Crataegus* species. Our results favor recognizing *Mespilus* and *Crataegus* as one genus. Molecular dating and biogeographic analyses showed that *Crataegus* originated in Southwest China during the early Oligocene, approximately 30.23 Ma ago. Transoceanic migration of East Asian *Crataegus* species across the Bering land bridge led to the development of North American species, whereas westward migration of the ancestors of *C. songarica* drove the formation of European species. *C. cuneata* may represent the earliest lineage of Chinese *Crataegus*. The uplift of the Qinghai–Tibet Plateau (QTP) and the Asian monsoon system may have led the ancestors of *C. cuneata* in south-western China to migrate toward the northeast, giving rise to other Chinese *Crataegus* species. This study offers crucial insights into the origins of *Crataegus* and proposes an evolutionary model for the genus.

## 1. Introduction

Hawthorn (*Crataegus* L.), widely distributed throughout temperate regions of the Northern Hemisphere, belongs to the genus *Crataegus* in the family Rosaceae [1]. The hawthorn fruit is widely used for its sweet and sour flavor, as well as its nutritional properties; it is favored by consumers because of its freshness, ease of processing, and medicinal properties [2,3]. Hawthorn has garnered significant global interest as an exceptional medicinal tonic due to its abundance of biologically active compounds [4,5]. In addition, hawthorns have significant ecological and ornamental value; their fruits serve as a food source for birds and mammals, while their dense branches offer ideal nesting locations for birds [6,7].

Phipps [8] suggested that *C. scabrifolia* of Southwest China and *C. mexicana* of Mexico are among the most primitive species and are similar to each other based on cladistic analysis. The evolution of the extant species began with *C. scabrifolia*, which spread into western Eurasia (one major line) and eastern Asia through Beringia to initiate a North American diversification (a minor line), as well as a minor North American diversification from *C. mexicana*. Owing to molecular and morphological characters, other researchers consider *Crataegus* as part of the subtribe Pyrinae and suggested that it originated in North America [9]. The relationships among species from eastern Asia, North America, and Europe were examined using sequences of chloroplast (cp) DNA regions and internal transcribed spacers (ITSs). The results indicated that North America and Europe might be the most recent areas of origin for *Crataegus* [10]. Generally, the origin of *Crataegus* remains unclear, and information about its evolutionary process is incomplete; thus, further research is required. In addition, a consensus is lacking on the relationship between *Mespilus* (medlar) and *Crataegus* (hawthorn), which are sister genera in the tribe Pyreae of the Rosaceae [11].

China is one of the places of origin of wild and cultivated *Crataegus* species, with a total of 18 species and six varieties [12]; however, others recognize 20 species of Chinese *Crataegus* and seven varieties across China [13]. Previous studies provide insight into the phylogenetic history of cultivated *Crataegus* in China. To date, there seem to be a lack of consensus on the phylogeny, taxonomy, origins and evolution of *Crataegus*. This hypothesis is in agreement with the study of Du et al. [14]. Based on specific-locus amplified fragment sequencing (SLAF-seq), Du et al. [14] suggested that the south-western Chinese species *C. scabrifolia* was the earliest Chinese species to separate and that this species shares a gene pool with European *Crataegus* species. However, others hold different views. Lo et al. [10] also proposed that North America and Europe are likely to be the most recent areas of origin for *Crataegus* based on sequencing of chloroplast (cp) DNA regions and internal transcribed spacers (ITSs); their findings indicated that *Crataegus* migrated from Europe to eastern Asia and then gave rise to *C. hupehensis*, *C. songorica* and *C. pinnatifida*. The ITS regions encompass ITS1, 5.8S rDNA and ITS2 spacer. ITS1 is located between 18S rDNA and 5.8S rDNA, whereas ITS2 is located between 5.8S rDNA and 28S rDNA. The intragenomic variability of the ITS2 region in *Crataegus* has negative consequences for DNA barcoding [15,16]. The length of this ITS in *Crataegus* is 675 bp. Its divergence among *Crataegus* species in east Asia, eastern North America and western North America is 0–0.85%, 0–2.3% and 0.41–1.72%, respectively [10].

A consensus is lacking on the relationship between *Mespilus* (medlar) and *Crataegus* (hawthorn), which are sister genera in the tribe Pyreae of the Rosaceae [11]. The genus *Mespilus* was considered to be a new, monotypic section in *Crataegus* based on two nuclear (ribosomal ITS and LEAFY intron 2) and four intergenic chloroplast DNA regions (trnS-trnG, psbA-trnH, trnH-rpl2 and rpl20-rps12) by Lo et al. [11]. However, Phipps [17] supports the existence of two genera according to morphological and cladistic evidence. Chloroplast genome sequences have been widely used to study plant evolutionary relationships at almost all taxonomic levels due to their maternal inheritance characteristic, lack of recombination, low nucleotide substitution rates and haploidy [18,19].

Genomic information for *Crataegus* is currently scarce, which complicates research pertaining to the taxonomy, genetics, identification and conservation of *Crataegus* species. Valuable information is contained in cp genome sequences, which permits the taxonomic classification and phylogenetic reconstruction of plant species and individuals [16,20]. Compared with nuclear genomes, cp genomes are much smaller in size and have high copy numbers per cell, which enables their complete sequencing [21]. The typical cp genomes in angiosperms usually have a circular structure containing a large single-copy (LSC) and a small single-copy (SSC) region separated by two inverted repeats (IRs) [22,23]. The rapid development of next-generation sequencing technologies has permitted a faster and more frugal cp genome sequencing. Complete cp genome analysis has been effectively employed in taxonomic and phylogenetic analyses, thus improving our understanding of evolutionary relationships, especially at lower taxonomic levels at which rapid radiation and recent speciation hamper the examination of sequence variations using conventional methods [24,25].

Here, the overall cp genomes of 19 *Crataegus* species (including 14 *Crataegus* species native to China, 2 *Crataegus* species from Europe, and 3 *Crataegus* species from North America) and 1 *Mespilus* species from Europe were sequenced using next-generation sequencing. For phylogenomic analysis, molecular dating and biogeographic analysis, the entire cp genomes of two published *Crataegus* species from the GenBank database, i.e., *C. marshallii* (MK920293.1) and *Crataegus* sp. BBL (MK920293.1), were obtained. The main objectives of this study were to analyze common structures and patterns of the *Crataegus* cp genomes; detect regional hotspots of sequences divergence; reconstruct the phylogenetic relationships among *Crataegus* species native to China; elucidate the relationship between *Crataegus* (hawthorn) and *Mespilus* (medlar); and determine the origin and evolution of the genus *Crataegus*. Our results will enable species identification in *Crataegus*, as well as support research in phylogenetic, phylogeographic and population genetic, which will in turn give a hand in the conservation and use of *Crataegus* resources.

## 2. Materials and Methods

### 2.1. Plant Material and DNA Extraction

Our sampling aimed to maximize the taxonomic and geographical coverage of *Crataegus*. Based on a preliminary field investigation and literature review, we finally chose 14 species native to China. Five exotic *Crataegus* species were chosen with respect to four major geographical areas (Eastern and Central North America, Eastern and Southern North America, Southeastern United States, and Europe). One *Mespilus* species was included, as only a single species is comprised in *Mespilus*.

Leaf material from the 14 Chinese *Crataegus* species was sampled from the National Germplasm Repository for *Crataegus* in Shenyang, Liaoning Province, China, while the leaves of the 5 exotic *Crataegus* species and 1 *Mespilus* species were sampled from Shanghai Chenshan Botanical Garden in Shanghai, China. The specific biogeographic regions and sampling locations of the 19 *Crataegus* species and 1 *Mespilus* species are displayed in Table 1. The CTAB method was used to extract DNA [26].

### 2.2. Chloroplast Genome Sequencing, Assembly and Annotation

Genomic DNA was fragmented to 400 bp fragments by ultrasound. Then, total DNA was purified and end-repaired. A 400 bp (insert size) paired-end library was constructed using a NEBNext Ultra DNA Library Prep Kit (New England Biolabs, Ipswich, MA, USA), and PE150 sequencing (Illumina, Inc., San Diego, CA, USA) was performed on an Illumina high-throughput sequencing platform (HiSeq 4000) (Illumina, Inc., San Diego, CA, USA). After sequencing, the raw reads were assembled into whole chloroplast genomes in a four-step approach. Firstly, adaptors were removed, and low-quality sequences were trimmed using Trimmomatic 0.39 [27] with the following parameters: LEADING = 20, TRAILING = 20, SLIDINGWINDOW = 4:15, MINLEN = 36, and AVGQUAL = 20. Secondly, the paired-end reads were evaluated for quality and assembled using SPAdes 3.6.1 (Kmer = 95) [28]. Thirdly, the assembled plastome sequences were selected from the initial assembly by a Basic Local Alignment Search Tool (BLAST)-based algorithm [29] using the chloroplast genome of *C. hupehensis* (MW201730) as a reference. Sequencher 4.10 (https://www.genecodes.com/) software was used to assemble selected contigs. At last, the command line Perl script Plann was used to annotate the *Crataegus* cp genomes [30]. Intron positions, putative starts and stops and initial annotation were determined with the *C. hupehensis* (MW201730) cp genome as a reference. Subsequently, Genome Vx was used to generate a physical map of the cp genome [31].

### 2.3. Molecular Marker Identification and Sequence Divergence Analysis

MAFFT v7 was employed to align the 20 cp genome sequences with the default parameters [32]. The nucleotide variability of the chloroplast genome was determined via sliding window analysis in DnaSP version 6.0 [33]. The analysis was conducted with a window size of 600 bp, and the step size was 100 bp. MEGA 7.0 software was employed to assess the variable and parsimony-informative base sites across the whole cp genomes and in the LSC, SSC and IR regions of the 20 cp genomes [34]. The p-distance among *Crataegus* plastomes was determined to assess the divergence pattern of the *Crataegus* species with MEGA software.

### 2.4. Phylogenetic Analysis

We subjected the 20 cp genome sequences generated, along with 2 whole cp genome sequences of *Crataegus* and the sequences of another 10 plastomes from the species of Maloideae downloaded from Genbank, to phylogenetic analysis. The maximum-likelihood (ML) and Bayesian inference (BI) methods were used to construct phylogenetic trees. Modelfinder was used to identify the optimal model GTR + F + I + G4 on the basis of the Bayesian information criterion [35]. ML analysis was performed with IQ-TREE 2.2.0 software [36], and sampling was repeated 1000 times. BI analysis was performed in the MrBayes program. The BI model was constructed, with ngen set to 1,000,000 burnin = ngen × 0.25/sample freq., Lset nst = 6 rates = invgamma, Prset statefreqpr = dirichlet (1, 1, 1, 1); graphical visualization of the STRUCTURE results was performed using Clumpak. Markov chain Monte Carlo (MCMC) analyses were performed for 10,000,000 generations. The trees were sampled every 1000 generations, and the first 25% sampled generations were discarded as burn-in samples. Finally, the average and standard deviation of split frequencies >0.01 were verified.

### 2.5. Divergence Time Estimation

Divergence time estimation was implemented using a relaxed log-normal clock model run on the BEAST v2.6.7 platform and a general time-reversible (GTR) substitution model and speciation tree prior based on the Yule speciation process [37]. We used one fossil constraint and one secondary calibration to estimate the divergence time: the stem of *Amelanchier* was dated to 33.9 Ma ago based on fossils of *A. peritula* and *A. scudderi* [38], and the estimated divergence date (50.06 Ma ago) of clade Maleae was used as the secondary calibration point [39].

BEAST analysis was executed for 100 million generations, sampling every 1000 generations. Using Tracer v1.6, the stationary phase was examined in terms of convergence and to secure an adequate sample size (>200) for all parameters. The initial 10% of trees were discarded (burn-in period). Subsequently, we used TreeAnnotatorv2.4.8 to summarize the time-calibrated species as a maximum clade credibility (MCC) tree [40]. For constructing the MCC tree, we set the credible set to 95%. This means that the top 95% of trees with the highest posterior probabilities were selected.

### 2.6. Ancestral Area Reconstruction

To rebuild the broad-scale biogeographic history of *Crataegus*, the distribution of each extant *Crataegus* species was encoded as a character with seven states based on the floristic regions reported by Zhao et al. [12] and Dong et al. [13] (Table 1) as follows: A, South-western China; B, Central Plains and Qinling Mountains of China; C, North-eastern China; D, Mongolia–Siberian region; E, Central and Western Asia; F, Europe; G, North America. We estimated the ancestral areas based on the collapsed MCC tree from the entire plastome datasets using the dispersal–extinction–cladogenesis (DEC) model, which was performed in RASP 4.0 [41]. Regarding the DEC analysis, the initial dispersal probabilities between areas were set as equal, with all entries in the dispersal constraint matrix assigned a value of 1. Only the most likely status (MLS) at the center of the pie diagram for each node is displayed. For detailed setting parameters, refer to Wang et al. [42]. The global temperature variation over the past 30 Ma was gained from Zachos et al. [43].

## 3. Results

### 3.1. Basic Characteristics of the Chloroplast Genome

**Figure 1:**
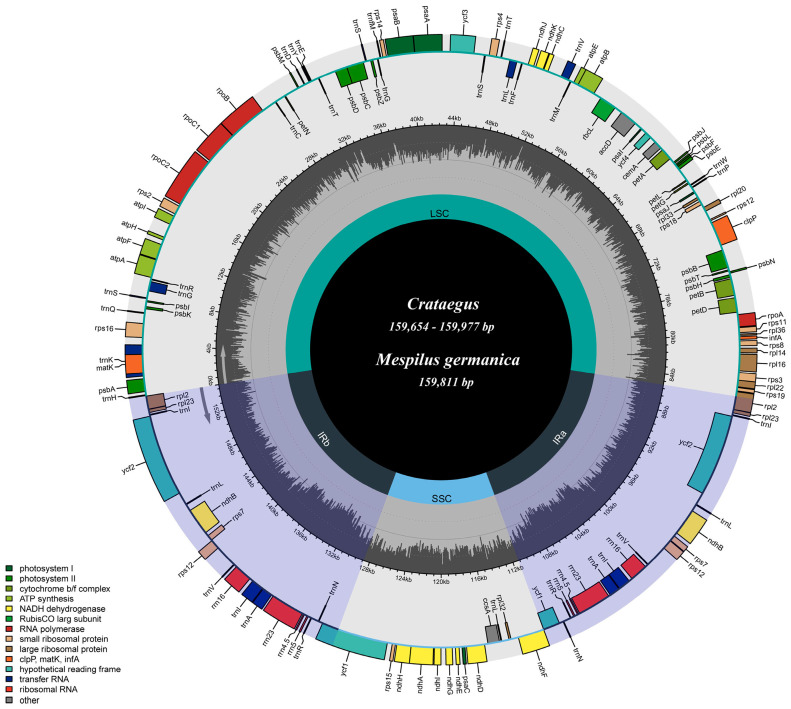
General chloroplast genome map of 19 *Crataegus* and 1 *Mespilus* species. The species sizes for the chloroplast genomes are shown in Table 2. Genes located inside the circle are transcribed in a clockwise direction, whereas those outside are transcribed counterclockwise. Genes are color-coded according to their function categories. The dashed area in the inner circle represents the GC content, while the lighter gray area indicates the AT content.

We submitted overall 20 complete cp genome sequences to the GenBank database with accession numbers MW201730 and OP963999~OP964017. The lengths of the nucleotide sequences of the 19 *Crataegus* cp genomes and 1 *Mespilus* cp genome ranged from 159,654 bp in *C. viridis* to 159,977 bp in *C. crussgalli* (Figure 1 and Table 2). All 20 cp genomes exhibited the typical quadripartite structure characteristic of angiosperms, including a pair of IRs (26,331–26,396 bp) separated by LSC (87,682–88,061 bp) and SSC (19,139–19,312 bp) regions. The overall GC content of the 20 cp genomes was 36.6–36.7%, with similar GC contents among all 20 genomes.

### 3.2. Sequence Divergence and Hotspots

We aligned the complete cp genome sequences of the 19 *Crataegus* species and 1 *Mespilus* species to determine sequence variations (Table 4). The alignment matrix spanned 163,856 bp in size, with 1115 variable sites (0.68%) including 654 parsimony-informative sites (0.4%) and 461 singleton sites (0.28%). The overall nucleotide diversity among the 20 cp genome sequences was 0.0018. The IR regions exhibited the lowest nucleotide sequence diversity (0.00032); that of the SSC regions was the highest (0.00355).

**Figure 2:**
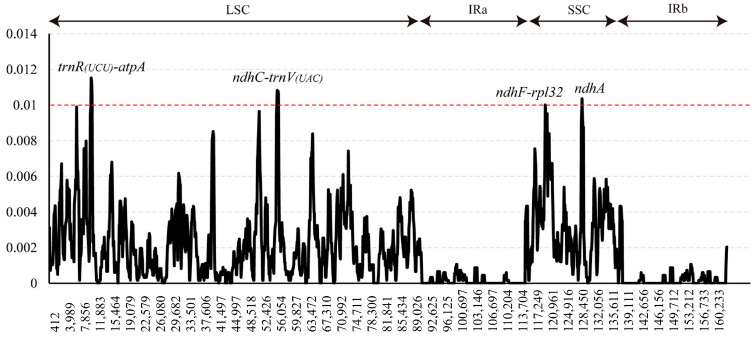
Sliding-window analysis of nucleotide variability across the 20 newly sequenced entire chloroplast genomes (window length: 600 bp; step size: 100 bp). *x*-Axis: position of the midpoint of each window; *y*-axis: nucleotide diversity of each window. The red dashed line means nucleotide diversity value is 0.01. The regions over the red dashed line mean they were characterized by high variability and high polymorphism. LSC, large single-copy; IRa, inverted repeat a; SSC, small single-copy; IRb, inverted repeat b.

To pinpoint sequence divergence hotspots, the nucleotide diversity (π) value was assessed within a sliding window of 600 bp (Figure 2). The π values ranged from 0 to 0.012. Four highly variable regions (π > 0.01), including *trnR(UCU)-atpA*, *ndhC-trnV(UAC)*, *ndhF-rpl32* and *ndhA*, were found in the *Crataegus* and *Mespilus* cp genomes (Figure 2). The *trnR(UCU)-atpA* region had the highest π values (π = 0.012), followed by the *ndhC-trnV(UAC), ndhA* and *ndhF-rpl32* regions. Among these sequences, *trnR(UCU)-atpA* and *ndhC-trnV(UAC)* are located in the LSC region, while *ndhF-rpl32* and *ndhA* are located in the SSC region. Meanwhile, the four hypervariable markers and universal DNA barcodes (*rbcL*, *matK* and *trnH-psbA*) were compared in greater detail (Table 5). The count of variable sites across the four marker regions ranged from 22 (*ndhC-trnV(UAC))* to 36 (*trnR(UCU)-atpA*), and the number of universal DNA barcode regions ranged from 8 (*rbcL* and *trnH-psbA*) to 16 (*matK*). Nucleotide diversity was much higher in the two major mutation hotspots (*trnR(UCU)-atpA* and *ndhC-trnV(UAC)*) than in the typical universal cp DNA barcode regions (*matK*, *rbcL* and *trnH-psbA*).

### 3.3. Phylogenetic Analysis

**Figure 3:**
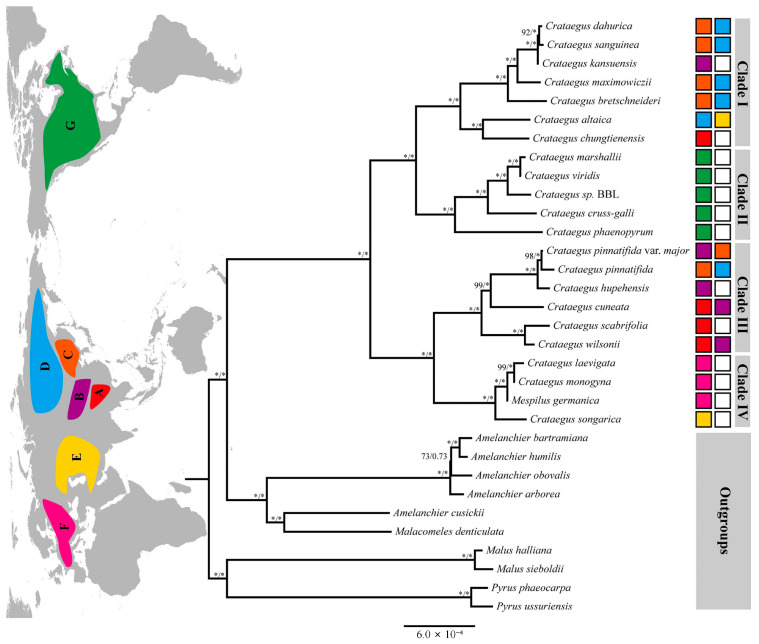
Phylogenetic analysis of *Crataegus* from maximum-likelihood analyses using complete chloroplast genomes. The four major Clades (I–IV) in the ingroup are represented by vertical bars, with the blocks on the right corresponding to the distribution of each species. The map indicates the species distribution of *Crataegus* used in the analysis. Seven regions were defined: (A) South-western China; (B) Central Plains and Qinling Mountains of China; (C) North-eastern China; (D) Mongolia–Siberian region; (E) Central and Western Asia; (F) Europe; (G) North America. The first and second numbers on the branches correspond to ML bootstrap support (BS) and Bayesian posterior probability (PP) values, respectively. The asterisks (*) indicate BS = 100% or PP = 1.0.

We conducted a phylogenetic analysis using 22 entire cp genomes obtained from 21 *Crataegus* species and 1 *Mespilus* species, with cp genomes of 10 other Maloideae species as the outgroup. As shown in Figure 3, the topologies of the ML and BI phylogenetic trees were generally consistent, showing two major clusters. *Crataegus* was closely related to the genus *Amelanchier*. Additionally, *Crataegus* and *Mespilus* formed a strongly supported (100%) monophyletic group.

Four well-supported clades (I–IV) were revealed within the ingroup based on a complete cp genome analysis, which successfully elucidated the species relationships within the clades (Figure 3). Apparently, these four clades clustered into two large lineages, in which Clades I and II were sister clades, as were Clades III and IV. Clade I consists of six sympatric or parapatric Chinese species (*C. dahurica*, *C. sanguinea*, *C. kansuensis*, *C. maximowiczii*, *C. bretschneideri* and *C. altaica*) found in the Mongolia–Siberian region, north-eastern China, and Central Plains and Qinling Mountains of China, along with one species from south-western China (*C. chungtienensis*). Clade II contained five North American species (*C. marshallii*, *C. viridis*, *C. sp*. BBL 2019, *C. crussgalli*, *C. phaenopyrum*). Clade III consisted of six Chinese species, i.e., *C. pinnatifida, C. pinnatifida* var*. major, C. hupehensis, C. cuneata, C. scabrifolia* and *C. wilsonii*, with distribution ranges including south-western, central, northern, eastern, and north-eastern China, as well as the Mongolia–Siberian region. In Clade IV, two European species (*C. laevigata* and *C. monogyna*) fell into a common subclade, with one Chinese species (*C. songarica*) found in Central and Western Asia forming a sister subclade.

### 3.4. Divergence Time Estimation

**Figure 4:**
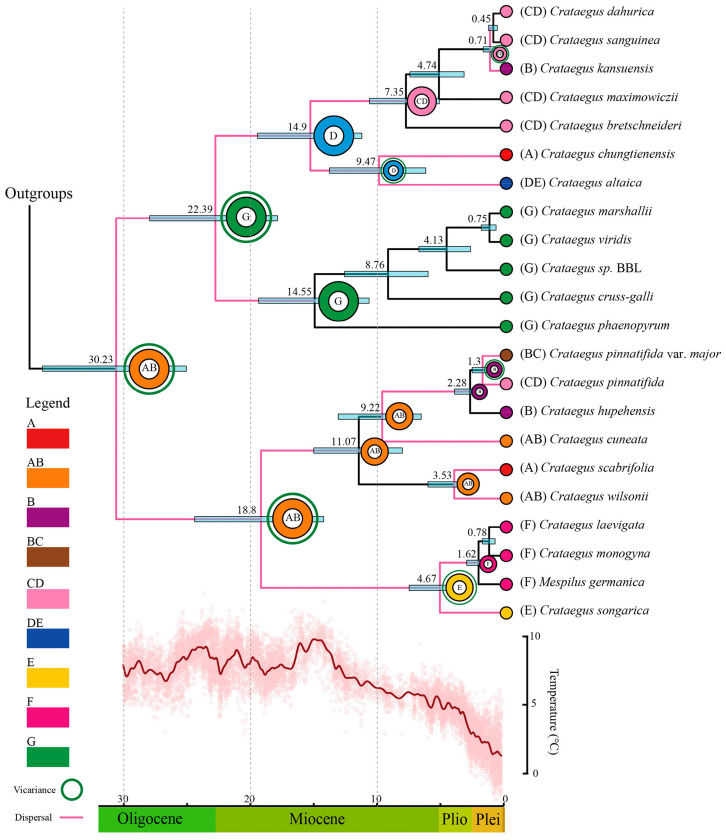
ML phylogenetic tree reconstruction of *Crataegus* species obtained from BEAST 2 analysis, with ancestral area reconstruction performed using the DEC model in RASP. The divergence times were estimated using a single fossil calibration (see text for more details). The branches are annotated with the most recent common ancestor (MRCA) times and their corresponding 95% highest posterior density (HPD) intervals (in Ma); cyan bars represent the 95% HPD intervals for node ages. Seven regions were defined: (A) South-western China; (B) Central Plains and Qinling Mountains of China; (C) North-eastern China; (D) Mongolia–Siberian region; (E) Central and Western Asia; (F) Europe; (G) North America. The letters and colors in the legend denote extant ancestral areas, as well as their combinations. The pie charts at each node illustrate the most probable ancestral areas.

On the basis of the complete cp genome datasets and fossil calibration, divergence time analysis showed that *Crataegus* originated and the split between Clades I + II and Clades III + IV occurred during the early Oligocene, approximately 30.23 Ma ago (Figure 4). The differentiation between Clades I and II was estimated to have occurred at approximately 22.39 Ma ago, while the split between Clade III and IV was estimated at 18.8 Ma ago. Early predecessors of the four clades began to emerge during the Miocene, with rapid divergence happening from the late Miocene to the Pliocene. In Clade I, the first Chinese *Crataegus* species to split were *C. altaica* and *C. chungtienensis,* which diverged around 9.47 Ma ago, followed by *C. bretschneideri* and *C. maximowiczii*, which split around 7.35 and 4.74 Ma ago, respectively, while *C. kansuensis, C. sanguinea* and *C. dahurica* successively split approximately 0.71 Ma and 0.45 Ma ago. Species diversification in Clade II was gradual, with *C. phaenopyrum* and *C. cruss-galli* splitting approximately 14.55 and 8.76 Ma ago, respectively, whereas divergence among *C. sp.* BBL, *C. viridis* and *C. marshallii* happened from 4.13 to 0.75 Ma ago. In Clade III, *C. cuneata,* which diverged around 9.22 Ma ago, was the first *Crataegus* to diverge, followed by *C. scabrifolia* and *C. wilsonii*, which split approximately 3.53 Ma ago. Divergence among *C. hupehensis*, *C. pinnatifida* and *C. pinnatifida* var. *major* emerged recently (4.14–0.75 Ma ago). In Clade IV, the intercontinental split between Asian and European species happened around 4.67 Ma ago, and divergence between *M. germanica* and European *Crataegus* occurred approximately 1.62 Ma ago. The split between *C. monogyna* and *C. laevigata* was estimated to have occurred 0.78 Ma ago.

### 3.5. Biogeographic History

Southwest China, the Central Plains and the Qinling Mountains of China are the most likely ancestral areas of *Crataegus*, according to the results of the DEC analysis (Figure 4). The current distribution of *Crataegus* appears to be shaped by a combination of dispersal and vicariance events. Integrating the results of the DEC analysis and molecular dating, we identified a long-distance dispersal route across the Bering land bridge from East Asia to North America (AB–G), which led to the divergence of the North American species *C. phaenopyrum* in the middle Miocene (approximately 14.55 Ma ago). Another long-distance spreading route across the Bering land bridge in the opposite direction (G–D–C) facilitated the occurrence of *C. altaica* and other species in north-eastern China (Clade I). The third long-distance spreading from East Asia to the Europe (AB–E–F) gave rise to speciation of the Chinese species *C. songarica* and the European species *Crataegus* in the early Pliocene (approximately 4.67 Ma ago) and Pleistocene (1.62–0.78 Ma ago), respectively. Within China, two important dispersals (A–B; B–C) and subsequent vicariance resulted in the colonization of Southwest China, the Central Plains, Qinling Mountains and Northeast China by *Crataegus*.

## 4. Discussion

### 4.1. Features of the Crataegus and Mespilus Chloroplast Genomes

In the present study, we sequenced the cp genomes of 19 *Crataegus* species and 1 *Mespilus* species using next-generation sequencing methods. The sizes of the 20 newly sequenced cp genome ranged in length from 159,654 bp (*C. viridis*) to 159,977 bp (*C. crussgalli*). All species contained a total of 113 genes, including 79 protein-coding genes, 30 tRNA genes and 4 rRNA genes. The genes *ycf15* and *ycf68* are not annotated due to their status as pseudogenes, which contain several internal stop codons [18]. Additionally, the genes *ycf2*, *rpl23* and *accD* are missing from the plastomes in some species [22,44,45]; on the contrary, these genes generally appear in *Crataegus*. The structures of the 20 newly sequenced cp genome gained in present study are generally analogous to those found in most plants, and no recombination events were found. The lowest sequence divergence was observed in IR regions, while all high-variation regions are located in the LSC region (Table 4); this finding is consistent with recent studies [18,23]. The most possible reason for this difference is that, in the cp genome, which is present in multiple copies within individual plant cells, gene conversion, which exhibits a minor bias against new mutations, is more effective at reducing the mutation load in the two IR regions than in the single-copy regions, primarily due to the duplicative inherent quality of the IRs [46,47,48,49]. The high-variation regions could be utilized to develop DNA barcoding methods for the estimation of plant phylogeny at low taxonomic levels, such as among species of *Crataegus.*

### 4.2. Potential Highly Variable Chloroplast Barcodes

A growing body of research shows that universal DNA barcodes (*rbcL, matK* and *trnH-psbA)* have low sequence divergence and do not offer a clear resolution of the phylogenetic relationship [28,50]. A few nuclear and cp DNA barcodes have been applied for the genetic characterization of *Crataegus* and to determine its phylogeny, taxonomy, origins and evolution [10,51,52]. Due to the formation of apomictic complexes, active introgression events and relatively rare segregation events related to older hybridizations, DNA barcoding and the taxonomic assessment of the genus *Crataegus* are challenging [8]. Hence, it is essential to utilize additional markers and expand the taxonomic sampling to achieve better resolution at low taxonomic levels. The hawthorn fruit is widely used for its sweet and sour flavor, as well as its nutritional properties [3]; however, genomic resources for *Crataegus* are scarce, which is a serious barrier to the progress of its taxonomy, genetics, conservation and utilization. Further study can be conducted to determine its genomic evolution and produce valuable genetic resources with the help of cp genome sequences. Mutation events in the cp genome may not have occurred randomly throughout the genome sequence; instead, they are often focused in hotspot regions [53]. An efficient means for detecting mutation hotspots is comparing cp genome sequences; these identified highly variable regions can be used as specific DNA markers. In the present study, four hypervariable regions, including *trnR(UCU)-atpA*, *ndhC-trnV(UAC)*, *ndhF-rpl32* and *ndhA* (Figure 2), were identified.

The *trnR(UCU)-atpA* region is the most variable region in the 20 cp genomes (Figure 2). It is part of the *trnG-atpA* intergenic marker, which is split between two intergenic regions: *trnG-trnR* and *trnR-atpA*. The *trnG-atpA* region could serve as a high-variability marker in *Corylus* [54]. *ndhC-trnV(UAC)* is part of the *ndhC-trnV(UAC)-trnM(CAU)* region and has been reported as a high-variability marker that can be used for DNA barcoding and molecular phylogenetic studies [55]. The *ndhF* and *ndhF-rpl32* regions are considered divergence hotspots by Wu et al. [56], who compared nine *Crataegus* cp genomes and identified five highly variable markers. These two regions, *ndhF* and *ndhF-rpl32*, have been widely used in plant phylogenetic studies [50,57].

### 4.3. Phylogenetic Relationships

As China is one of the places of origin of wild and cultivated *Crataegus* species, the collection and cultivation of hawthorns has been documented in China for >1000 years [12]. Although a total of 18 species and six varieties have been identified and confirmed [58], the phylogenetic relationships among *Crataegus* species native to China remain undetermined.

*Crataegus* is a genus of the Maloideae subfamily in the Rosaceae [59]. The taxonomy of the genus *Crataegus* is complicated by a mix of biological and historical factors. Frequent interspecific hybridization, widespread polyploidy and apomixis can all obscure the distinctions between *Crataegus* species [60,61]. The genetic relationships among some species of *Crataegus* have been identified using DNA markers such as simple sequence repeats (SSRs), cpSSRs (non-coding cp regions), restriction fragment length polymorphism (RFLP) and ITS [6,7,10,62]. However, these molecular markers suggest that considerable information is missing. In recent years, the cp genome has become an effective strategy to elucidate phylogenetic relationships at almost all taxonomic levels [17,63]. Wu et al. [64] published a phylogenetic study of Chinese *Crataegus* based on cp genome sequences; however, in that study, only 4 of 18 sequences were derived from whole-genome assemblies. In our study, the newly sequenced cp genomes of 20 species (including 14 *Crataegus* species native to China, 2 *Crataegus* species from Europe, 3 *Crataegus* species from North America and 1 *Mespilus* species from Europe), and two whole cp genome sequences of *Crataegus* obtained from GenBank were analyzed comparatively; the higher coverage in this study may shed more light on the interspecific relationships among *Crataegus* species native to China.

A well-supported phylogenetic tree for 14 Chinese *Crataegus* species was constructed, according to the complete cp genome sequences (Figure 3). The ML phylogenetic tree showed that 14 Chinese *Crataegus* species clustered into two clades (Clade I and Clade III, Figure 3). Based on morphological characters, Chinese *Crataegus* species can be divided into four groups [12], which form two clades derived from the cp genomes. Clade I consists of *C. sanguinea, C. kansuensis, C. dahurica, C. maximowiczii*, *C. bretschneideri*, *C. altaica* and *C. chungtienensis*, while Clade III comprises *C. pinnatifida, C. pinnatifida* var. *major*, *C. hupehensis*, *C. cuneata*, *C. scabrifolia*, *C. wilsonii* and *C. songarica*. From a geographic perspective, Clade I is a northern group except for *C. chungtienensis*, whereas Clade III is widely distributed, with species ranges in south-western, central, northern, eastern, and north-eastern China. Clade I and the North American *Crataegus* species grouped together, as did Clade III and the European *Crataegus* species. These results provide evidence for a genetic exchange between Chinese and North American or European species of *Crataegus*, consistent with previous studies [8,10,14]. In Clade I, *C. sanguinea, C. kansuensis* and *C. dahurica* clustered together, with very little difference seen among the three species. In accordance with our results, Wu et al. [65] and Zhang et al. [62] implied that *C. sanguinea, C. kansuensis* and *C. dahurica* are closely related based on RFLP and SSR analyses.

As noted above, Clade I is a northern group except for *C. chungtienensis*. *C. chungtienensis* is predominantly found in alpine areas of Southwest China, at elevations of around 2500–3300 m [12], whereas *C. altaica* is endemic to north-western China. However, *C. chungtienensis* and *C. altaica* grouped together. The variation among the cp sequences aligned with the geographic distribution of each species, indicating that repeated cp DNA introgression resulted in the present distribution [66]. Hybridization and introgression are effective means to generate new species in plant evolution, or ecotypes, and drive reticulate evolution [15,67,68]. The cytoplasm of one species might be replaced by that of another via gene flow infiltration through hybridization and repeated backcrossing. Consequently, the genetic components of one species includes not only the nuclear genome inherited from its parents but also newly acquired cp gene sequences. Introgression-induced cp capture events have been commonly reported in sympatric and parapatric plant taxa [54,68,69,70]. Multiple cp capture events within *Crataegus* may have been enabled by frequent colonization events associated with the long-distance dispersal of fruits by birds. The spread of *C. chungtienensis* and *C. altaica* has likely relied on birds. The pomes of *Crataegus*, as well as those of other berry-like fruits approximately 1 cm in size, are commonly considered as bird-dispersed fruits [8,64,71].

Research on the relationship between *Crataegus* (hawthorn) and *Mespilus* (medlar) has been ongoing for a long time, but controversies persist, and researchers have not yet reached a consensus regarding these two genera. The longstanding consensus among academic taxonomists is that *Mespilus* and *Crataegus* are distinct genera [9,59]. Two nuclear and four intergenic cp DNA regions were used to assess the relationship between *Mespilus* and *Crataegus*; the results did not confirm a distinction between the genera *Mespilus* and *Crataegus* [11]. Talent et al. [60] proposed conserving the name *Crataegus* rather than *Mespilus*. However, Phipps [17] contested that morphological and cladistic evidence favors the recognition of two distinct genera. *Mespilus* and *Crataegus* are treated as two distinct genera by some researchers [38,72]. In the present study, *Mespilus germanica* fell within the genus *Crataegus* in the phylogenetic tree based on complete cp genome sequences (Figure 3). Furthermore, *Mespilus germanica* and the two European *Crataegus* species grouped together, and *C. songarica* was the sister group. Consequently, our results favor the recognition of *Mespilus* and *Crataegus* as one genus.

### 4.4. Origin and Spread of Crataegus

The current biogeographic patterns and species speciation processes in the northern temperate zone are the result of both geological events and long-term climate changes emerging since the Tertiary [69,73]. Molecular dating and biogeographic analysis (Figure 4) showed that *Crataegus* emerged around 30.23 Ma ago, which is adjacent to the era of the Eocene–Oligocene climate transition (approximately 34 Ma ago) [74]. Therefore, the early development of *Crataegus* is related to the temperature. As the temperature in high-latitude regions (45–70 degrees in both hemispheres) decreased during the Eocene–Oligocene climate transition [74], *Crataegus* became distributed in Southwest China, as well as in the Central Plains and Qinling Mountains of China. Southwest China, ranging from the eastern Himalayas and Yarlung Zangbo Canyon to the entire Hengduan Mountains, is recognized as one of thirty-four biodiversity hotspots by Conservation International [70]. This region is considered the place of origin of the genus *Crataegus*, consistent with previous biogeographic studies [8].

On the origin and spread of *Crataegus*, there are many hypotheses. Phipps [8] postulated that *Crataegus* migrated eastward from East Asia to North America via the Bering land bridge in the early Tertiary or Miocene, as well as westward from Southwest China to Europe. Nevertheless, other researchers have divergent views. Evans and Campbell [9] proposed that *Crataegus* originated in North America. Lo et al. [10] posited that *Crataegus* originated in Europe and eastern North America and that the Chinese *Crataegus* species *C. songarica, C. pinnatifida* and *C. hupehensis* derived from the European *Crataegus* that spread into eastern Asia. Our results showed three intercontinental migrations based on ancestral area reconstruction (Figure 4). One migratory pathway involved East Asian *Crataegus* species migrating to North America across the Bering land bridge 30.23–22.39 Ma ago. The opposite dispersal route, along which *Crataegus* migrated westward from North America to East Asia across the Bering land bridge, was active 22.39–14.9 Ma ago. The Bering land bridge made a significant contribution to the interchange between Asia and North American biotas and is therefore globally regarded as a key area for paleogeographic and biogeographic research [75]. A possible explanation regarding the disjunct distributions of related extant flora in eastern Asia and western North America is spreading across the Bering land bridge [76]. Dispersal from Asia into North America reached a peak during the late Oligocene warming period (26–24 Ma ago), while influx from North America into Asia emerged at a lower rate than migration in the opposite direction throughout the Cenozoic, with peak rates of dispersal occurring in the early to middle Miocene (16–14 Ma ago) [76]. Along the third intercontinental migratory pathway, Chinese *Crataegus* in East Asia migrated to Central Asia 18.8–4.67 Ma ago, resulting in the speciation of *C. songarica*, which migrated further into Europe 4.67–0.78 Ma ago and led to the formation of the *M. germanica* and European *Crataegus* species. The uplift of the Qinghai–Tibet Plateau (QTP) was a pivotal geological event in the Cenozoic era. The analysis of magnetic susceptibility and soil particle size in the north-eastern area of the QTP indicated a significant uplift 7–8 and 2.6 Ma ago [74,77]. The geography and climate of Asia significantly changed due to the uplift of the QTP, which drives the formation of the East Asian winter monsoon system [78]. The Asian monsoon system leads to precipitation occurring mainly at low latitudes, leaving the mid-latitude areas of Asia in arid conditions [64]. Due to these events, the ancestors of *C. songarica* in north-western China migrated westward to Europe.

In addition to these three intercontinental migrations, the results of ancestral area reconstruction also suggested two other important dispersals (A–B; B–C) (Figure 4). Du et al. [14] suggested that a divergence event for *Crataegus* species native to China happened in south-western China and led to their migration northward and eastward to north-eastern China. *C. scabrifolia* in south-western China, which was determined as the earliest-diverging Chinese species, progressed toward the northeast and split to form *C. pinnatifida* [14]. However, according to the results of our molecular dating analysis (Figure 4), *C. cuneata* may represent the earliest lineage of *Crataegus*. *C. cuneata*, which is widespread in the warm temperate and south-eastern regions of China, split approximately 9.22 Ma ago. Based on the results of ancestral area reconstruction (Figure 4), the ancestor of *C. cuneata* is likely to have migrated northward and eastward, splitting to form *C. scabrifolia, C. hupehensis, C. pinnatifida* and *C. pinnatifida* var. *major.* This migratory route is consistent with a migration pathway from the Hengduan Mountains north-eastward through the Qinling Range, i.e., the eastern fringe of the Loess Plateau including the Taihang Range, Yinshan Range and Changbai Mountains, as suggested by Wang [79] based on the distribution patterns and migration routes of some East Asian flora. An intense uplift of the QTP occurred three times during the Cenozoic era [80]. The uplift of the QTP significantly altered the climate in Asia and resulted in the formation of the Asian monsoon system [81,82]. As a result, precipitation in south-western China is not well distributed, which leads to the migration of plants [83,84]. Together, these climatic events may have prompted the ancestors of *C. cuneata* in south-western China to migrate toward the northeast.

The present study provides the complete cp genomes of 19 *Crataegus* species and 1 *Mespilus* species and sheds light on the phylogeny, taxonomy, origins and evolution of *Crataegus*. However, the limited number of species analyzed in the present study may have resulted in possible errors in the phylogenetic analysis. In future studies, a higher number of species and more extensive nuclear gene information should be included.

## 5. Conclusions

In this study, the complete cp genomes of 19 *Crataegus* species and 1 *Mespilus* species were sequenced. A comparative analysis of the plastomes led to the identification of four hypervariable hotspots, *trnR(UCU)-atpA*, *ndhC-trnV(UAC)*, *ndhF-rpl32* and *ndhA*, which could serve as molecular markers for the genus *Crataegus*. In subsequent studies, species of hawthorn can be identified by designing primers to further determine whether the hypervariable hotspots can be used for identification.

A phylogenetic analysis based on the complete cp genome revealed that the 14 Chinese *Crataegus* species examined could be divided into two clades to help elucidate the phylogenetic relationships among *Crataegus* species in China. One clade and the North American *Crataegus* species grouped together, while the other clade grouped with European *Crataegus* species. Additionally, our findings support the recognition of *Mespilus* and *Crataegus* as one genus.

Molecular dating and biogeographic analyses indicated that *Crataegus* originated in Southwest China during the early Oligocene, approximately 30.23 Ma ago. The transoceanic migration of east Asian *Crataegus* species across the Bering land bridge led to the development of North American species, whereas westward migration of the ancestors of *C. songarica* drove the formation of European species. The uplift of the Qinghai–Tibet Plateau (QTP) and the Asian monsoon system may have led the ancestors of *C. cuneata* in south-western China to migrate toward the northeast, giving rise to other Chinese *Crataegus* species. Overall, the present study provides valuable information about the phylogeny, taxonomy, origins and evolution of *Crataegus*.

## Figures and Tables

**Table 1:** Biogeographic regions, sample collection sites and related information of 19 *Crataegus* species and 1 *Mespilus* species.

Taxon	Identification Code	Biogeographic Region	Collection Site	Usage	Main Characteristics
*C. bretschneideri*	FSZ	North-eastern China, Mongolia–Siberia	Shenyang	Fresh eating, processing, medicine, ornamental plant, afforestation	Fruit, small, prismatic and oblate, peel, bright red
*C. pinnatifida*	SLH	North-eastern China, Mongolia–Siberia	Shenyang	Medicine, ornamental plant, afforestation	Fruit, subglobose or pear-shaped, peel, dark red
*C. pinnatifida* var*. major*	SDDJX	Central Plains, Qinling Mountains of China and North-eastern China	Shenyang	Fresh eating, processing, medicine, afforestation	Fruit, large, broad obovate, peel, dark red or purple red
*C. maximowiczii*	MSZ	North-eastern China, Mongolia–Siberia	Shenyang	Fresh eating, ornamental plant, afforestation, timber	Fruit, spherical, pilose when young, glabrous after, peel, red
*C. hupehensis*	HBSZ1H	Central Plains and Qinling Mountains of China	Shenyang	Fresh eating, processing, medicine, ornamental plant, afforestation	Fruit, small, subglobose, peel, bright red
*C. sanguinea*	LNSZ	North-eastern China, Mongolia–Siberia	Shenyang	Fresh eating, medicine, ornamental plant, afforestation	Fruit, extremely small, oblate, peel, blood red and smooth
*C. kansuensis*	GSSZ	Central Plains and Qinling Mountains of China	Shenyang	Fresh eating, processing, medicine, afforestation	Fruit, subspheroidal, peel, red or orange
*C. scabrifolia*	YNSZ	South-western China	Shenyang	Fresh eating, processing, medicine	Fruit, medium size, oblate, peel, stone yellow
*C. songarica*	ZGESZ	Central and Western Asia	Shenyang	Medicine, ornamental plant	Fruit, spherical or oblate, peel, reddish black
*C. cuneata*	XYSZ	Southwest, Central Plains and Qinling Mountains of China	Shenyang	Processing, medicine, rootstock	Fruit, spherical or oblate, peel, red or yellow
*C. altaica*	AETSZ	Central and Western Asia, Mongolia–Siberia	Shenyang	Medicine, ornamental plant	Fruit, small and spherical, peel, golden yellow
*C. dahurica*	GYSZ	North-eastern China, Mongolia–Siberia	Shenyang	Fresh eating, medicine, afforestation, rootstock	Fruit, spherical or oblong, peel, orange-red or orange
*C. chungtienensis*	ZDSA	South-western China	Shenyang	Ornamental plant, afforestation	Fruit, small, spherical or oblong, peel, red
*C. wilsonii*	HZSZ	Southwest, Central Plains and Qinling Mountains of China	Shenyang	Processing, afforestation	Fruit, small, oblong, peel, red
*C. crussgalli*	JJSZ	North America	Shanghai	Ornamental plant, afforestation	Thorn, strong, fruit, small, peel, red
*C. viridis*	LSZ	North America	Shanghai	Ornamental plant, afforestation	Fruit, small, peel, bright red
*C. phaenopyrum*	HSDSZ	North America	Shanghai	Ornamental plant, afforestation	Fruit, small, peel, bright red
*C. laevigata*	HHSZ	Europe	Shanghai	Ornamental plant	Fruit, scarce, peel, dark red
*C. monogyna*	DZSZ1H	Europe	Shanghai	Ornamental plant, afforestation	dunga-runga, peel, dark red
*M. germanica*	OZ	Europe	Shanghai	Fresh eating, processing, afforestation	Dunga-runga, fruit, apple-shaped, peel, brown

**Table 2:** General characteristics of the 20 newly sequenced chloroplast genomes.

Species	Plastome Size (bp)	LSC Length (bp)	IR Length (bp)	SSC Length (bp)	Gene Number	Protein-Coding Genes	tRNA Genes	rRNA Genes	Overall GC (%)	GenBank Accession Number
*C* *. pinnatifida*	159,676	87,744	26,384	19,164	113	79	4	30	36.60%	OP963999
*C. pinnatifida* var. *major*	159,656	87,749	26,384	19,139	113	79	4	30	36.70%	OP964000
*C* *. maximowiczii*	159,800	87,778	26,384	19,254	113	79	4	30	36.60%	OP964001
*C* *. cuneata*	159,718	87,766	26,384	19,184	113	79	4	30	36.60%	OP964002
*C* *. sanguinea*	159,851	87,844	26,384	19,239	113	79	4	30	36.60%	OP964003
*C* *. scabrifolia*	159,744	87,759	26,383	19,219	113	79	4	30	36.60%	OP964004
*C* *. songarica*	159,871	87,880	26,389	19,213	113	79	4	30	36.60%	OP964005
*C* *. kansuensis*	159,827	87,800	26,384	19,259	113	79	4	30	36.60%	OP964006
*C* *. altaica*	159,704	87,682	26,377	19,268	113	79	4	30	36.60%	OP964007
*C* *. bretschneideri*	159,717	87,711	26,347	19,312	113	79	4	30	36.60%	OP964008
*C* *. dahurica*	159,852	87,847	26,384	19,237	113	79	4	30	36.60%	OP964009
*C* *. chungtienensis*	159,835	87,803	26,384	19,264	113	79	4	30	36.60%	OP964010
*C* *. laevigata*	159,707	87,698	26,384	19,241	113	79	4	30	36.60%	OP964011
*C* *. monogyna*	159,763	87,779	26,384	19,216	113	79	4	30	36.60%	OP964012
*C* *. hupehensis*	159,766	87,852	26,385	19,144	113	79	4	30	36.60%	MW201730
*C* *. wilsonii*	159,779	87,773	26,383	19,240	113	79	4	30	36.60%	OP964013
*C* *. crussgalli*	159,977	88,014	26,363	19,237	113	79	4	30	36.60%	OP964014
*C* *. phaenopyrum*	159,953	88,061	26,331	19,230	113	79	4	30	36.60%	OP964015
*C* *. viridis*	159,654	87,704	26,365	19,220	113	79	4	30	36.60%	OP964016
*M* *. germanica*	159,811	87,804	26,396	19,215	113	79	4	30	36.60%	OP964017

Note: LSC, large single-copy; SSC, small single-copy; IR, inverted repeat in the cp genomes of *Crataegus* and *Mespilus;* a total of 113 genes were identified, containing 79 protein-coding genes, 4 ribosomal RNA (rRNA) genes, and 30 transfer RNA (tRNA) genes (Figure 1 and Table 3). Eight protein-coding genes (*ndhB*, *rpl2*, *rpl23*, *rps7*, *rps12*, *rps19*, *ycf1* and *ycf2*), seven tRNA genes (*trnI-CAU*, *trnL-CAA*, *trnV-GAC*, *trnI-GAU*, *trnA-UGC*, *trnR-ACG* and *trnN-GUU*), and all four rRNA genes are duplicated in IR regions (Figure 1 and Table 3). Sixteen genes include introns, with two genes (*clpP* and *ycf3*) having two introns each, while the remaining fourteen genes (*atpF*, *petB, petD, ndhA*, *ndhB*, *rps12*, *rpl2*, *rpl16*, *trnA-UGC*, *trnG-GCC*, *trnI-GAU*, *trnK-UUU*, *trnL-UAA* and *trnV-UAC*) each contain one intron (Figure 1 and Table 3). Notably, *rps12* is trans-spliced, with its 5′ exon located in the LSC region, and its 3′ exon and intron duplicated and located in the IR regions (Figure 1). The *trnK-UUU* gene has the longest intron, which contains the *matK* gene.

**Table 3:** Gene composition of the 20 newly sequenced chloroplast genomes.

Gene Category	Gene Group	Gene Names
Photosynthesis-related genes	Rubisco	*rbcL*
	Photosystem I	*psaA*, *psaB*, *psaC*, *psaI*, *psaJ*
	Assembly/stability of photosystem I	* ycf3, *ycf4*
	Photosystem II	*psbA*, *psbB*, *psbC*, *psbD*, *psbE*, *psbF*, *psbH*, *psbI*, *psbJ*, *psbK*, *psbL*, *psbM*, *psbN*, *psbT*, *psbZ*
	ATP synthase	*atpA*, *atpB*, *atpE*, * atpF, *atpH*, *atpI*
	Cytochrome complex	*petA*, * petB, * petD, *petG*, *petL*, *petN*
	Cytochrome synthesis	*ccsA*
	NADPH dehydrogenase	* ndhA, * ndhB, *ndhC*, *ndhD*, *ndhE*, *ndhF*, *ndhG*, *ndhH*, *ndhI*, *ndhJ*, *ndhK*
Transcription- and translation-related genes	Transcription	*rpoA*, *rpoB*, *rpoC1*, *rpoC2*
	Ribosomal proteins	*rps2*, *rps3*, *rps4*, *rps7*, *rps8*, *rps11*, * rps12, *rps14*, *rps15*, *rps16*, *rps18*, *rps19*, * rpl2, *rpl14*, * rpl16, *rpl20*, *rpl22*, *rpl23*, *rpl32*, *rpl33*, *rpl36*
	Translation initiation factor	*infA*
RNA genes	Ribosomal RNA	*rrn5*, *rrn4.5*, *rrn16*, *rrn23*
	Transfer RNA	* trnA-UGC, *trnC-GCA*, *trnD-GUC*, *trnE-UUC*, *trnF-GAA*, *trnfM-CAU*, *trnG-UCC*, * trnG-GCC, *trnH-GUG*, *trnI-CAU*, * trnI-GAU, * trnK-UUU, *trnL-CAA*, * trnL-UAA, *trnL-UAG*, *trnfM-CAUI*, *trnM-CAU*, *trnN-GUU*, *trnP-UGG*, *trnQ-UUG*, *trnR-ACG*, *trnR-UCU*, *trnS-GCU*, *trnS-GGA*, *trnS-UGA*, *trnT-GGU*, *trnT-UGU*, *trnV-GAC*, * trnV-UAC, *trnW-CCA*, *trnY-GUA*
Other genes	RNA processing	*matK*
	Carbon metabolism	*cemA*
	Proteolysis	* clpP
	Fatty acid synthesis	*accD*
Genes of unknown function	Conserved reading frames 2	*ycf1*, *ycf2*

Intron-containing genes are marked with asterisks (*).

**Table 4:** Results of variable-site analysis of the 20 newly sequenced chloroplast genomes.

Region	Length (bp)	Variable Sites		Parsimony- Informative Sites		Singleton Sites		Nucleotide Diversity
		Number	%	Number	%	Number	%	
LSC	91,171	803	0.88	461	0.51	342	0.38	0.00232
SSC	19,881	254	1.28	159	0.80	95	0.48	0.00355
IR	26,402	29	0.11	17	0.06	12	0.05	0.00032
Total	163,856	1115	0.68	654	0.40	461	0.28	0.00180

**Table 5:** The variability of four highly variable markers and universal chloroplast DNA barcodes (*rbcL*, *matK* and *trnH-psbA*) was investigated in *Crataegus* and *Mespilus*.

Marker	Length (bp)	Variable Sites		Parsimony- Informative Sites		Nucleotide Diversity
		Number	%	Number	%	
*trnR(UCU)-atpA*	847	36	4.25	17	2.01	0.01381
*ndhC-trnV(UAC)*	758	22	2.90	12	1.58	0.01227
*ndhF-rpl32*	1221	31	0.56	18	0.49	0.00732
*ndhA*	2310	26	1.05	17	0.66	0.00352
*trnH-psbA*	297	8	4.25	5	2.01	0.00781
*rbcL*	1428	8	2.90	7	1.58	0.00188
*matK*	1521	16	2.54	10	1.47	0.00293

## Data Availability

The complete chloroplast genomes generated during the current study were submitted to the NCBI database and are available with the GenBank accession numbers MW201730 and OP963999~OP964017.

## References

[1] Christensen K.I. (1992). Revision of Crataegus sect. Crataegus and Nothosect. Crataeguineae (Rosaceae-Maloideae) in the old world. Syst. Bot. Monogr..

[2] Özcan M., Hacıseferoğulları H., Marakoğlu T., Arslan D. (2005). Hawthorn (*Crataegus* spp.) fruit: Some physical and chemical properties. J. Food Eng..

[3] Xu J.Y., Zhao Y.H., Zhang X., Zhang L.J., Hou Y.L., Dong W.X. (2016). Transcriptome analysis and ultrastructure observation reveal that hawthorn fruit softening is due to Cellulose/Hemicellulose degradation. Front. Plant Sci..

[4] Liu P., Yang B., Kallio H. (2010). Characterization of phenolic compounds in Chinese hawthorn (*Crataegus pinnatifida* Bge. Var. *Major*) fruit by high performance liquid chromatography-electrospray ionization mass spectrometry. Food Chem..

[5] Zheng G., Deng J., Wen L., You L., Zhao Z., Zhou L. (2018). Release of phenolic compounds and antioxidant capacity of Chinese hawthorn “*Crataegus pinnatifida*” during in vitro digestion. J. Func. Foods.

[6] Brown J.A., Beatty G.E., Finlay C., Montgomery W.I., Tosh D.G., Provan J. (2016). Genetic analyses reveal high levels of seed and pollen flow in hawthorn (*Crataegus monogyna* Jacq.), a key component of hedgerows. Tree Genet. Genomes.

[7] Betancourt-Olvera M., Nieto-Angel R., Urbano B., Gonzalez-Andres F. (2018). Analysis of the biodiversity of hawthorn (*Crataegus* spp.) from the morphological, molecular, and ethnobotanical approaches, and implications for genetic resource conservation in scenery of increasing cultivation: The case of Mexico. Genet. Resour. Crop Evol..

[8] Phipps J.B. (1983). Biogeographic, taxonomic, and cladistic relationships between East Asiatic and North American Crataegus. Ann. Mo. Bot. Gard..

[9] Evans R.C., Campbell C.S. (2002). The origin of the apple subfamily (Maloideae; Rosaceae) is clarified by DNA sequence data from duplicated GBSSI genes. Am. J. Bot..

[10] Lo E.Y.Y., Stefanović S., Christensen K.I., Dickinson T.A. (2009). Evidence for genetic association between East Asian and western North American Crataegus L. (Rosaceae) and rapid divergence of the eastern North American lineages based on multiple DNA sequences. Mol. Phylogenet. Evol..

[11] Lo E.Y.Y., Stefanović S., Dickinson T.A. (2007). Molecular reappraisal of relationships between *Crataegus* and *Mespilus* (Rosaceae, Pyreae)–Two genera or one?. Syst. Bot..

[12] Zhao H.C., Feng B.T. (1996). China Fruit-Plant Monograph of Hawthorn (Crataegus) Flora.

[13] Dong W.X., Li Z.X. (2015). The Science and Practice of Chinese Fruit Tree: Hawthorn.

[14] Du X., Zhang X., Bu H., Zhang T., Lao Y., Dong W. (2019). Molecular Analysis of Evolution and Origins of Cultivated Hawthorn (*Crataegus* spp.) and Related Species in China. Front. Plant Sci..

[15] Zarrei M., Stefanovic’ S., Dickinson T.A. (2014). Reticulate evolution in North American black-fruited hawthorns (*Crataegus* section Douglasia; Rosaceae): Evidence from nuclear ITS2 and plastid sequences. Ann. Bot..

[16] Park I., Choi B., Weiss-Schneeweiss H., So S., Myeong H.H., Jang T.S. (2022). Comparative Analyses of Complete Chloroplast Genomes and Karyotypes of Allotetraploid *Iris koreana* and Its PutativeDiploid Parental Species (*Iris* Series *Chinenses*, Iridaceae). Int. J. Mol. Sci..

[17] Phipps J.B. (2016). Studies in *Mespilus*, *Crataegus*, and ×*Crataemespilus* (Rosaceae), I. Differentiation of *Mespilus* and *Crataegus*, expansion of ×*Crataemespilus*, with supplementary observations on differences between the *Crataegus* and *Amelanchier* clades. Phytotaxa.

[18] Lu R.S., Li P., Qiu Y.X. (2017). The complete chloroplast genomes of three *cardiocrinum* (Liliaceae) species: Comparative genomic and phylogenetic analyses. Front. Plant Sci..

[19] Roy N.S., Jeong U., Na M., Choi I., Cheong E.J. (2020). Genomic analysis and a consensus chloroplast genome sequence of *Prunus yedoensis* for DNA marker development. Hortic. Environ. Biotechnol..

[20] Liu Q., Li X., Li M., Xu W., Heslop-Harrison J.S. (2020). Comparative chloroplast genome analyses of *Avena*: Insights into evolutionary dynamics and phylogeny. BMC Plant Biol..

[21] McNeal J.R., Leebens-Mack J.H., Arumuganathan K., Kuehl J., Boore J.L., DePamphilis C.W. (2006). Using partial genomic fosmid libraries for sequencing complete organellar genomes. Biotechniques.

[22] Wicke S., Schneeweiss G.M., DePamphilis C.W., Muller K.F., Quandt D. (2011). The evolution of the plastid chromosome in land plants: Gene content, gene order, gene function. Plant Mol. Biol..

[23] Mehmetoglu E., Kaymaz Y., Ates D., Kahraman A., Tanyolac M.B. (2022). The complete chloroplast genome sequence of *Cicer echinospermum*, genome organization and comparison with related species. Sci. Hortic..

[24] Wang J., Li Y., Li C., Yan C., Shan S. (2019). Twelve complete chloroplast genomes of wild peanuts: Great genetic resources and a better understanding of *Arachis* phylogeny. BMC Plant Biol..

[25] Saina J.K., Gichira A.W., Li Z., Hu G., Wang Q., Liao K. (2018). The complete chloroplast genome sequence of *Dodonae aviscosa*: Comparative and phylogenetic analyses. Genetica.

[26] Li J.L., Wang S., Yu J., Wang L., Zhou S.L. (2013). A modified CTAB protocol for plant DNA extraction. Chin. Bull. Bot..

[27] Bolger A.M., Lohse M., Usadel B. (2014). Trimmomatic: A flexible trimmer for Illumina sequence data. Bioinformatics.

[28] Bankevich A., Nurk S., Antipov D., Gurevich A.A., Dvorkin M., Kulikov A.S., Lesin V.M., Nikolenko S.I., Pham S., Prjibelski A.D. (2012). SPAdes: A new genome assembly algorithm and its applications to single-cell sequencing. J. Comput. Biol..

[29] Brozynska M., Furtado A., Henry R.J. (2014). Direct chloroplast sequencing: Comparison of sequencing platforms and analysis tools for whole chloroplast barcoding. PLoS ONE.

[30] Huang D.I., Cronk Q. (2015). Plann: A command-line application for annotating plastome sequences. Appl. Plant Sci..

[31] Conant G.C., Wolfe K.H. (2008). GenomeVx: Simple web-based creation of editable circular chromosome maps. Bioinformatics.

[32] Katoh K., Standley D.M. (2013). MAFFT multiple sequence alignment software version 7: Improvements in performance and usability. Mol. Biol. Evol..

[33] Rozas J., Albert F.M., Juan Carlos S.D., Sara G.R., Pablo L., Ramos-Onsins S.E., Alejandro S.G. (2017). DnaSP 6: DNA Sequence Polymorphism Analysis of Large Data Sets. Mol. Biol. Evol..

[34] Kumar S., Stecher G., Tamura K. (2016). MEGA7: Molecular Evolutionary Genetics Analysis Version 7.0 for Bigger Datasets. Mol. Biol. Evol..

[35] Kalyaanamoorthy S., Minh B.Q., Wong T.K.F., von Haeseler A., Jermiin L.S. (2017). ModelFinder: Fast model selection for accurate phylogenetic estimates. Nat. Methods.

[36] Nguyen L.T., Schmidt H.A., von Haeseler A., Minh B.Q. (2015). IQ-TREE: A Fast and Effective Stochastic Algorithm for Estimating Maximum-Likelihood Phylogenies. Mol. Biol. Evol..

[37] Bouckaert R., Vaughan T.G., BaridoSottani J., Duchene S., Fourment M., Gavryushkina A., Heled J., Jones G., Kuhnert D., De Maio N. (2019). BEAST 2.5: An advanced software platform for Bayesian evolutionary analysis. PLoS Comput. Biol..

[38] Xiang Y.Z., Huang C.H., Hu Y., Wen J., Li S.S., Yi T.S., Chen H.Y., Xiang J., Ma H. (2017). Evolution of Rosaceae Fruit Types Based on Nuclear Phylogeny in the Context of Geological Times and Genome Duplication. Mol. Biol. Evol..

[39] Zhang S.D., Jin J.J., Chen S.Y., Chase M.W., Soltis D.E., Li H.T., Yang J.B., Li D.Z., Yi T.S. (2017). Diversification of Rosaceae since the Late Cretaceous based on plastid phylogenomics. New Phytol..

[40] Rambaut A., Drummond A.J. (2014). TreeAnnotator v2. 1.2.

[41] Yu Y., Harris A.J., Blair C., He X. (2015). RASP (Reconstruct Ancestral State in Phylogenies): A tool for historical biogeography. Mol. Phylogenet. Evol..

[42] Wang Y., Sun J., Qiao P., Wang J., Wang M., Du Y., Xiong F., Luo J., Yuan Q., Dong W. (2022). Evolutionary history of genus *Coptis* and its dynamic changes in the potential suitable distribution area. Front. Plant Sci..

[43] Zachos J.C., Dickens G.R., Zeebe R.E. (2008). An early Cenozoic perspective on greenhouse warming and carbon-cycle dynamics. Nature.

[44] Oliver M.J., Murdock A.G., Mishler B.D., Kuehl J.V., Boore J.L., Mandoli D.F., Everett K.D., Wolf P.G., Duffy A.M., Karol K.G. (2010). Chloroplast genome sequence of the moss *Tortula ruralis*: Gene content, polymorphism, and structural arrangement relative to other green plant chloroplast genomes. BMC Genom..

[45] Jansen R.K., Cai Z., Raubeson L.A., Daniell H., Depamphilis C.W., Leebens-Mack J., Kai F.M., Guisinger-Bellian M., Haberle R.C., Hansen A.K. (2007). Analysis of 81 genes from 64 plastid genomes resolves relationships in angiosperms and identifies genome-scale evolutionary patterns. Proc. Natl. Acad. Sci. USA.

[46] Perry A.S., Wolfe K.H. (2002). Nucleotide substitution rates in legume chloroplast DNA depend on the presence of the inverted repeat. J. Mol. Evol..

[47] Zhu A.D., Guo W.H., Gupta S., Fan W.S., Mower J.P. (2016). Evolutionary dynamics of the plastid inverted repeat: The effects of expansion, contraction, and loss on substitution rates. New Phytol..

[48] Li F.W., Kuo L.Y., Pryer K.M., Rothfels C.J. (2016). Genes translocated into the plastid inverted repeat show decelerated substitution rates and elevated GC content. Genome Biol. Evol..

[49] Wu C.S., Chaw S.M. (2015). Evolutionary stasis in cycad plastomes and the first case of plastome GC-Biased gene conversion. Genome Biol. Evol..

[50] Asaf S., Waqas M., Khan A.L., Khan M.A., Kang S.M., Imran Q.M., Shahzad R., Bilal S., Yun B.W., Lee I.J. (2017). The complete chloroplast genome of wild rice (*Oryza minuta*) and its comparison to related species. Front. Plant Sci..

[51] Emami A., Shabanian N., Rahmani M., Khadivi A., Mohammad-Panah N. (2018). Genetic characterization of the *Crataegus* genus: Implications for in situ conservation. Sci. Hortic..

[52] Erfani-Moghadam J., Mozafari M., Fazeli A. (2016). Genetic variation of some hawthorn species based on phenotypic characteristics and RAPD marker. Biotechnol. Biotechnol. Equip..

[53] Dong W.P., Liu J., Yu J., Wang L., Zhou S. (2012). Highly variable chloroplast markers for evaluating plant phylogeny at low taxonomic levels and for DNA barcoding. PLoS ONE.

[54] Hu G., Cheng L., Huang W., Cao Q., Zhou L., Jia W., Lan Y. (2020). Chloroplast genomes of seven species of Coryloideae (Betulaceae): Structures and comparative analysis. Genome.

[55] Raman G., Park K.T., Kim J.H., Park S.J. (2020). Characteristics of the completed chloroplast genome sequence of *Xanthium spinosum*: Comparative analyses, identification of mutational hotspots and phylogenetic implications. BMC Genom..

[56] Wu X., Luo D., Zhang Y., Yang C., Crabbe M.J.C., Zhang T., Li G. (2022). Comparative Genomic and Phylogenetic Analysis of Chloroplast Genomes of Hawthorn (*Crataegus* spp.) in Southwest China. Front. Genet..

[57] Yang Z., Zhao T., Ma Q., Liang L., Wang G. (2018). Comparative genomics and phylogenetic analysis revealed the chloroplast genome variation and interspecific relationships of *Corylus* (Betulaceae) species. Front. Plant Sci..

[58] Guo T.J., Jiao P.J. (1995). Hawthorn (*Crataegus*) resources in China. HortScience.

[59] Campbell C.S., Donoghue M.J., Wojciechowski B.M.F. (1995). Phylogenetic relationships in Maloideae (Rosaceae): Evidence from sequences of the internal transcribed spacers of nuclear ribosomal DNA and its congruence with morphology. Am. J. Bot..

[60] Talent N., Eckenwalder J.E., Lo E., Christensen K.I., Dickinson T.A. (2008). (1847) Proposal to conserve the name *Crataegus* against *Mespilus* (*Rosaceae*). Taxon.

[61] Dnmez E.O. (2008). Pollen morphology in Turkish *Crataegus* (Rosaceae). Bot. Helv..

[62] Zhang Y., Dai H.Y., Zhang Q.J., Li H., Zhang Z.H. (2008). Assessment of genetic relationship in *Crataegus* genus by the apple SSR primers. J. Fruit Sci..

[63] Sun J., Sun R., Liu H., Chang L., Li S., Zhao M., Shennan C., Lei J., Dong J., Zhong C. (2021). Complete chloroplast genome sequencing of ten wild *Fragaria* species in China provides evidence for phylogenetic evolution of *Fragaria*. Genomics.

[64] Wu L.W., Cui Y.X., Wang Q., Xu Z.C., Wang Y., Lin Y.L., Song J.Y., Yao H. (2021). Identification and phylogenetic analysis of five *Crataegus* species (Rosaceae) based on complete chloroplast genomes. Planta.

[65] Wu F.F., Zhang Z.H., Dai H.Y., Zhang Y., Chang L.L. (2008). Genetic Relationships of Some Hawthorns (*Crataegus* spp.) Derived from cp DNA PCR-RFL. J. Shenyang Agric. Univ..

[66] Fehrer J., Gemeinholzer B., Chrtek J.J., Brautigam S. (2007). Incongruent plastid and nuclear DNA phylogenies reveal ancient intergeneric hybridization in *Pilosella* hawkweeds (*Hieracium*, Cichorieae, Asteraceae). Mol. Phylogenet. Evol..

[67] Paun O., Forest F., Fay M.F., Chase M.W. (2009). Hybrid speciation in angiosperms: Parental divergence drives ploidy. New Phytol..

[68] Du F.K., Peng X.L., Liu J.Q., Lascoux M., Hu F.S., Petit R.J. (2011). Direction and extent of organelle DNA introgression between two spruce species in the Qinghai-Tibetan Plateau. New Phytol..

[69] Acosta M.C., Premoli A.C. (2010). Evidence of chloroplast capture in South American *Nothofagus* (subgenus *Nothofagus*, Nothofagaceae). Mol. Phylogenet. Evol..

[70] Zhao T., Wang G., Ma Q., Liang L., Yang Z. (2020). Multilocus data reveal deep phylogenetic relationships and intercontinental biogeography of the Eurasian-North American genus *Corylus* (Betulaceae). Mol. Phylogenet. Evol..

[71] Hokanson K.E., Smith M.J., Connor A.M., Luby J.J., Hancock J.F. (2006). Relationships among subspecies of New World octoploid strawberry species, *Fragaria virginiana* and *Fragaria chiloensis*, based on simple sequence repeat marker analysis. Can. J. Bot..

[72] Zarei A., Erfani-Moghadam J., Mozaffari M. (2017). Phylogenetic analysis among some pome fruit trees of Rosaceae family using RAPD markers. Biotechnol. Biotechnol. Equip..

[73] Xiang X.G., Wang W., Li R.Q., Lin L., and Liu Y., Zhou Z.K., Li Z.Y., Chen Z.D. (2014). Large-scale phylogenetic analyses reveal fagalean diversification promoted by the interplay of diaspores and environments in the Paleogene. Perspect. Plant Ecol. Evol. Syst..

[74] Liu Z.H., Pagani M., Zinniker D., DeConto R., Huber M., Brinkhuis H., Shah S.R., Leckie R.M., Pearson A. (2009). Global cooling during the Eocene-Oligocene climate transition. Science.

[75] Marincovich L., Gladenkov A.Y. (1999). Evidence for an early opening of the Bering Strait. Nature.

[76] Jiang D., Klaus S., Zhang Y.P., Hillis D.M., Li J.T. (2019). Asymmetric biotic interchange across the Bering land bridge between Eurasia and North America. Natl. Sci. Rev..

[77] Liu X., Mao X., Ding Z., Lv B., Chen F. (2009). Study on the relation between loess paleoclimate trend and uplift Tibetan Plateau. Quat. Sci..

[78] Gong H., Zhang Y., Huang L. (2005). Paleoenvironment significance of grain-size composition of Neogene Red Clay in Linxia Basin, Gansu province. Acta Sedimentol. Sin..

[79] Wang W.T. (1992). On some distribution patterns and some migration routes found in the eastern Asiatic region. Acta Phys. Sin..

[80] Lu H., Wang X., An Z., Miao X.D., Zhu R.X., Ma H.Z., Li Z., Tan H.B., Wang X.Y. (2004). Geomorphologic evidence of phased uplift of the northeastern Qinghai-Tibet Plateau since 14 million years ago. Sci. China Ser. D.

[81] Li J.J. (2006). The Qinghai-Tibet Plateau Uplifting and Environmental Evolution in Asia: Article Collection of Academician Li Ji-Jun.

[82] Liu X.D., Dong B.W. (2013). Influence of the Tibetan Plateau uplift on the Asian monsoon-arid environment evolution. Chin. Sci. Bull..

[83] Jacques F., Guo S.X., Tao S., Xing Y.W., Huang Y.J., Liu Y.S., Ferguson D.K., Zhou Z.K. (2011). Quantitative reconstruction of the Late Miocene monsoon climates of southwest China: A case study of the Lincang flora from Yunnan Province. Palaeogeogr. Palaeoclimatol. Palaeoecol..

[84] Su T., Liu Y.S., Jacques F., Huang Y.J., Xing Y.W., Zhou Z.K. (2013). The intensification of the East Asian winter monsoon contributed to the disappearance of *Cedrus* (Pinaceae) in southwestern China. Quat. Res..

